# Blocking core fucosylation of epidermal growth factor (EGF) receptor prevents peritoneal fibrosis progression

**DOI:** 10.1080/0886022X.2021.1918557

**Published:** 2021-05-17

**Authors:** Changqing Yu, Ning Yang, Weidong Wang, Xiangning Du, Qingzhu Tang, Hongli Lin, Longkai Li

**Affiliations:** Department of Nephrology, Liaoning Translational Medicine Center of Nephrology, First Affiliated Hospital of Dalian Medical University, Dalian, China

**Keywords:** Core fucosylation, epidermal growth factor receptor, peritoneal fibrosis, peritoneal dialysis

## Abstract

**Objective:**

Peritoneal fibrosis (PF) ultimately causes ultrafiltration failure and peritoneal dialysis (PD) termination, but there are few effective therapies for it. Core fucosylation, which is catalyzed by α1,6-fucosyltransferase (Fut8) in mammals, may play a crucial role in PF development. This study aims to assess the effects of inhibiting core fucosylation of epidermal growth factor (EGF) receptor on PF rats.

**Methods:**

PF rats (established by 4.25% glucose dialysate) were treated with either an adenovirus-Fut8 short hairpin RNA (Fut8shRNA) or adenovirus-control. Masson’s staining and net ultrafiltration were performed at week six. Fut8 level and core fucosylation of EGF receptor and collagen I in the peritoneal membrane were assessed, and EGF signaling was detected, including signal transducer and activator of transcription 3 (STAT3), nuclear factor kappa B (NF-κB) and their phosphorylation. Monocyte chemoattractant protein-1 (MCP-1) in peritoneal effluent was examined.

**Results:**

Fut8 was upregulated in PF rats but decreased after Fut8shRNA treatment. EGF and EGF receptor expression was upregulated in PF rats, while core fucosylation of EGF receptor decreased after Fut8shRNA treatment. Masson’s staining results showed an increase in peritoneal thickness in PF rats but a decrease after Fut8shRNA treatment. Fut8shRNA treatment increased net ultrafiltration, reduced the expression of collagen I and MCP-1 compared to PF rats. Fut8shRNA treatment suppressed phosphorylation of STAT3 and NF-κB in the peritoneal membrane of PF rats.

**Conclusions:**

Fut8shRNA treatment ameliorated the fibrotic changes in PF rats. A potential mechanism may be that Fut8shRNA treatment inactivated EGF signaling pathway by suppressing the phosphorylation of STAT3 and NF-κB.

## Introduction

Peritoneal dialysis (PD), a type of home dialysis therapy for end-stage renal disease, has been widely used across the world because of its convenience and flexibility [1], especially in the current COVID-19 pandemic [2]. However, long-term PD treatment will result in peritoneal fibrosis, a frequent complication due to exposure to glucose peritoneal dialysate [3,4]. Peritoneal fibrosis may affect the function of the peritoneal membrane and ultimately lead to ultrafiltration failure and discontinuation of PD [5,6]. Unfortunately, the mechanism of peritoneal fibrosis is not fully elucidated [7–9], and there are few effective methods available to treat peritoneal fibrosis; therefore, targets to prevent peritoneal fibrosis are urgently required.

Previous studies showed that multiple signaling pathways were involved in the progression of peritoneal fibrosis, including transforming growth factor-β1 (TGF-β1) [10,11], platelet-derived growth factor (PDGF) [7,12], and epidermal growth factor (EGF) pathways [13]. Peritoneal fibrosis was significantly attenuated by individually blocking the abovementioned signaling pathways [7,11,13]. However, as multiple signaling pathways have key effects in peritoneal fibrosis, blocking one signaling pathway may not suffice to prevent peritoneal fibrosis. With that in mind, we hypothesized that one potential strategy to provide stronger protection against peritoneal fibrosis was to simultaneously inhibit multiple signaling pathways. How to inhibit one target and block multiple signaling pathways? There is an urgent need to find such same key target in multiple signaling pathways.

Currently, posttranslational modifications of proteins mediate direct and definitive regulation of their functions and affect the activation of multiple signaling pathways [14,15], where TGF-β1, PDGF, and EGF pathway receptors are modified by core fucosylation that is catalyzed by α1,6-fucosyltransferase (Fut8) [16]. Our previous studies showed that inhibiting core fucosylation of TGF-β1 and PDGF receptors significantly ameliorated the progression of peritoneal fibrosis in rats [17]. However, other glycoproteins, such as the EGF receptor, are also modified by core fucosylation. It is unknown whether inhibiting core fucosylation of other glycoproteins may be beneficial in protecting the peritoneal membrane. A better understanding of the signaling pathways associated with core fucosylation of the key proteins is essential in the development of peritoneal fibrosis.

Since the EGF signaling pathway plays a crucial role in the progression of peritoneal fibrosis, and the EGF receptor has been reported to be modified by core fucosylation, we designed the present study to observe the effects of core fucosylation of EGF receptor on peritoneal fibrosis. We first investigated whether core fucosylation of the EGF receptor was involved in peritoneal fibrosis development in rats, and then observed the effect of inhibiting its core fucosylation on preventing peritoneal fibrosis.

## Materials and methods

### Animals

Adult male Sprague-Dawley (SD) rats (200–250 g) were obtained from the laboratory animal center of Dalian Medical University (Dalian, China). The animals were allowed to access food and water and housed with a 12 h light/dark cycle. The experiments were performed according to the Guide for the Care and Use of Laboratory Animals, which was published by the U.S. National Institutes of Health (NIH Publication No. 85-23, revised in 1996), and the experiments were also approved by the Ethics Committee of Dalian Medical University (approval number 8177032062).

### Construction of recombinant adenoviruses encoding rat Fut8shRNA

Since Fut8 is the unique fucosyltransferase responsible for core fucosylation, three Fut8 short hairpin RNAs (Fut8shRNA) were chemically synthesized and connected [14]. Then, a Fut8-knockdown rat model with peritoneal fibrosis was created, which has been described previously in more detail [17]. The recombinant adenovirus-control was also constructed following the same procedure [14].

### Experimental groups and treatment

The rat model of peritoneal fibrosis was established by a daily intraperitoneal injection of 4.25% glucose dialysate (Dianeal, Baxter) at 100 mL/kg body weight for six weeks, as previously reported [17,18]. Rats were randomly divided into four groups (*n* = 8 per group) and treated as follows: (1) the normal saline (NS) group received a daily injection of normal saline (100 mL/kg body weight); (2) the peritoneal fibrosis (PF) group received a daily infusion of 4.25% Dianeal; (3) the adenovirus-control (Ad-con) group received a single intraperitoneal injection of 1 × 10^9^ plaque-forming units (PFUs) of Ad-con (Genepharma, Wuhan, China), together with a daily infusion of 4.25% Dianeal; and (4) the adenovirus-Fut8 (Ad-Fut8) group received a single intraperitoneal injection of 1 × 10^9^ PFUs of Ad-Fut8shRNA (Genepharma), together with a daily infusion of 4.25% Dianeal. Parietal peritoneal membrane (opposite side to the injection point) was obtained at week six, fixed in 10% formalin, and embedded in paraffin.

## Fucosylation detection

Because Fut8 is the enzyme uniquely responsible for core fucosylation *in vivo,* Fut8 was examined to detect the presence of core fucose in rat peritoneal membranes by western blotting, according to our previously detailed protocol [15].

## Histology and immunohistochemistry

The formalin-fixed parietal peritoneal membrane was embedded in paraffin and prepared in sections that were 3 μm thick. Masson’s staining was performed for the evaluation of peritoneal fibrosis. Peritoneal thickness was measured, and an average of 10 independent measurements were calculated for each section (original magnification: × 100). For immunohistochemistry examination, the sections were deparaffinized, next endogenous peroxidase activity was quenched. After antigen retrieval, the sections were washed, incubated with primary antibodies and appropriate secondary antibodies, followed by treatment with a different reagent, which was described in our previous study [17]. The indexes for examination were collagen I, p-STAT3, and p-NF-κB. The immunohistochemical staining (brown) area in the peritoneum was quantitatively measured using Image-Pro Plus 6.0 software (Media Cybematics, Silver Spring, MD, USA).

## Peritoneal equilibrium test

A peritoneal equilibrium test was performed to assess the ultrafiltration function of peritoneal membrane at week six. Four hours after intraperitoneal administration of 4.25% Dianeal (100 mL/kg body weight), peritoneal cavity fluid was collected for ultrafiltration measurements. Net ultrafiltration was the result that the collected fluid volume after 4 h minus the administered fluid volume.

## Measurement of MCP-1

During peritoneal equilibrium test, peritoneal cavity fluid was collected and spun down to remove cellular debris, and then the supernatants were frozen at −80 °C until assay of monocyte chemoattractant protein-1 (MCP-1) by Enzyme-linked immunosorbent assay (ELISA) with ELISA kits (R&D System, Minneapolis, MN) as describled in our previous study [19].

## Immunoprecipitation

Immunoprecipitation was carried out according to our previously detailed protocol [14]. Briefly, peritoneal membrane was homogenized in cold RIPA lysis buffer, incubated and centrifuged. Lysates were precleared using Protein G PLUS-Agarose (Santa Cruz Biotechnology, CA), and then incubated with 2 mg of anti-EGF receptor antibody at 4 °C for 4 h on a rocker platform (30 rocks/min). Protein–antibody complexes were collected by adding Protein G PLUS-Agarose to each sample, which were incubated at 4 °C under rotary agitation overnight. After the beads were removed by washing, proteins were collected and stored for lectin blotting.

## Lectin blotting

Lectin blotting of the immunoprecipitates of EGF receptors were performed as detailed previously [14]. Briefly, polyvinylidene difluoride membranes were blocked with 100 mL of 5% bovine serum albumin (wt/vol) for 1 h at 25 °C with gentle agitation, and incubated for 2 h at 25 °C in Tris-buffered saline containing 0.05% Tween 20 (TBST) containing 1 mg/ml Lens culinaris agglutinin (LCA)-Biotin (Vector Labs), which preferentially recognizes Fuc-1,6GlcNAc. After washing with TBST four times for 10 min, lectin-reactive proteins were detected using an ECL kit.

## Western blotting

Western blotting was performed as previously described [14]. Fut8, EGF, EGF receptor, signal transducer and activator of transcription 3 (STAT3), nuclear factor kappa B (NF-κB), p-STAT3, and p-NF-κB were examined, and bands were detected using an ECL kit (Amersham-Pharmacia Biotech, Little Chalfont, UK). The protein expression was quantified using Labworks Image Analysis software (UVP, Upland, CA).

## Statistical analysis

Data examined in the experiments were expressed as the mean ± SEM values. Statistical analysis of data was performed between groups using a one-way ANOVA. Statistical significance was defined as *p* < 0.05.

## Results

### Fut8 was upregulated in the peritoneal membrane of rats with peritoneal fibrosis

**Figure 1.**
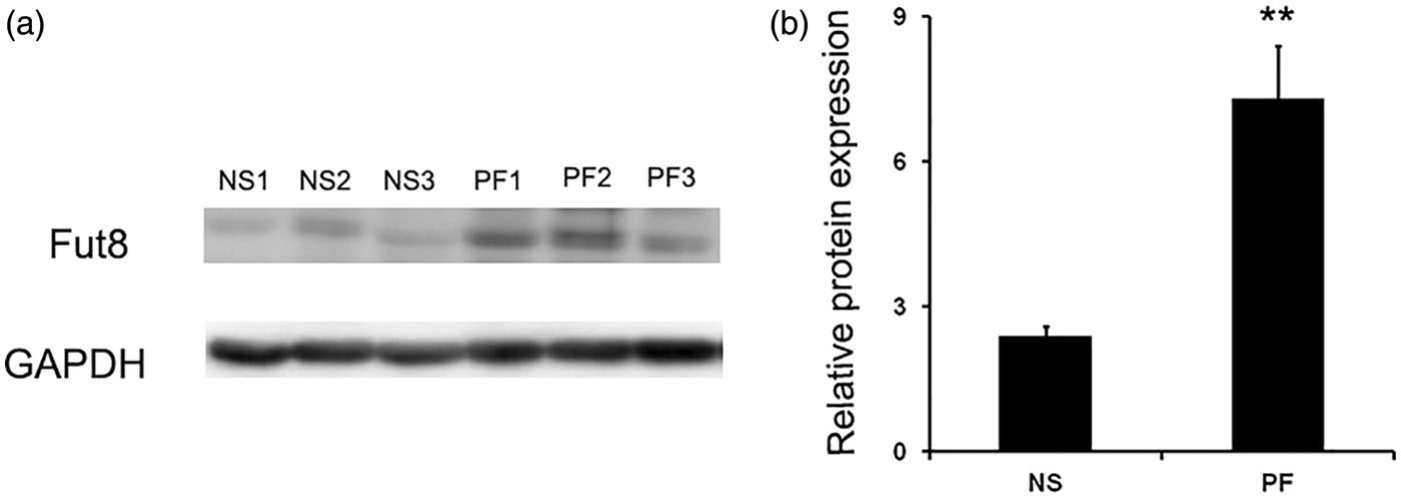
Fut8 expression was upregulated in the peritoneum of peritoneal fibrosis (PF) rats. PF rat model was established by daily intraperitoneal injection of standard peritoneal dialysis fluid for 6 weeks. (a and b) Representative Western blot images and analyses of Fut8 in normal saline (NS) and peritoneal fibrosis (PF) rats. Results are expressed as mean ± SEM of eight rats per group. **p < 0.01 vs. NS group.

An intraperitoneal injection with standard glucose PD dialysate for six weeks resulted in a typical fibrotic lesion in the peritoneal membrane of rats; and an increased peritoneal thickness was observed in rats with peritoneal fibrosis; peritoneal ultrafiltration rates dramatically decreased in rats with peritoneal fibrosis described previously [17]. The expression of collagen I by immunohistochemistry was dramatically increased after glucose PD dialysate intraperitoneal injection (supplementary Figure 1). All the changes confirmed a successful establishment of peritoneal fibrosis model in the present study. Since Fut8 is the enzyme responsible for core fucosylation *in vivo,* we assessed its expression in the peritoneal membrane of rats to determine whether it was associated with peritoneal fibrosis. Western blotting revealed that Fut8 was significantly upregulated in rats with peritoneal fibrosis (Figure 1(a,b)).

### EGF receptor was modified by core fucosylation

**Figure 2.**
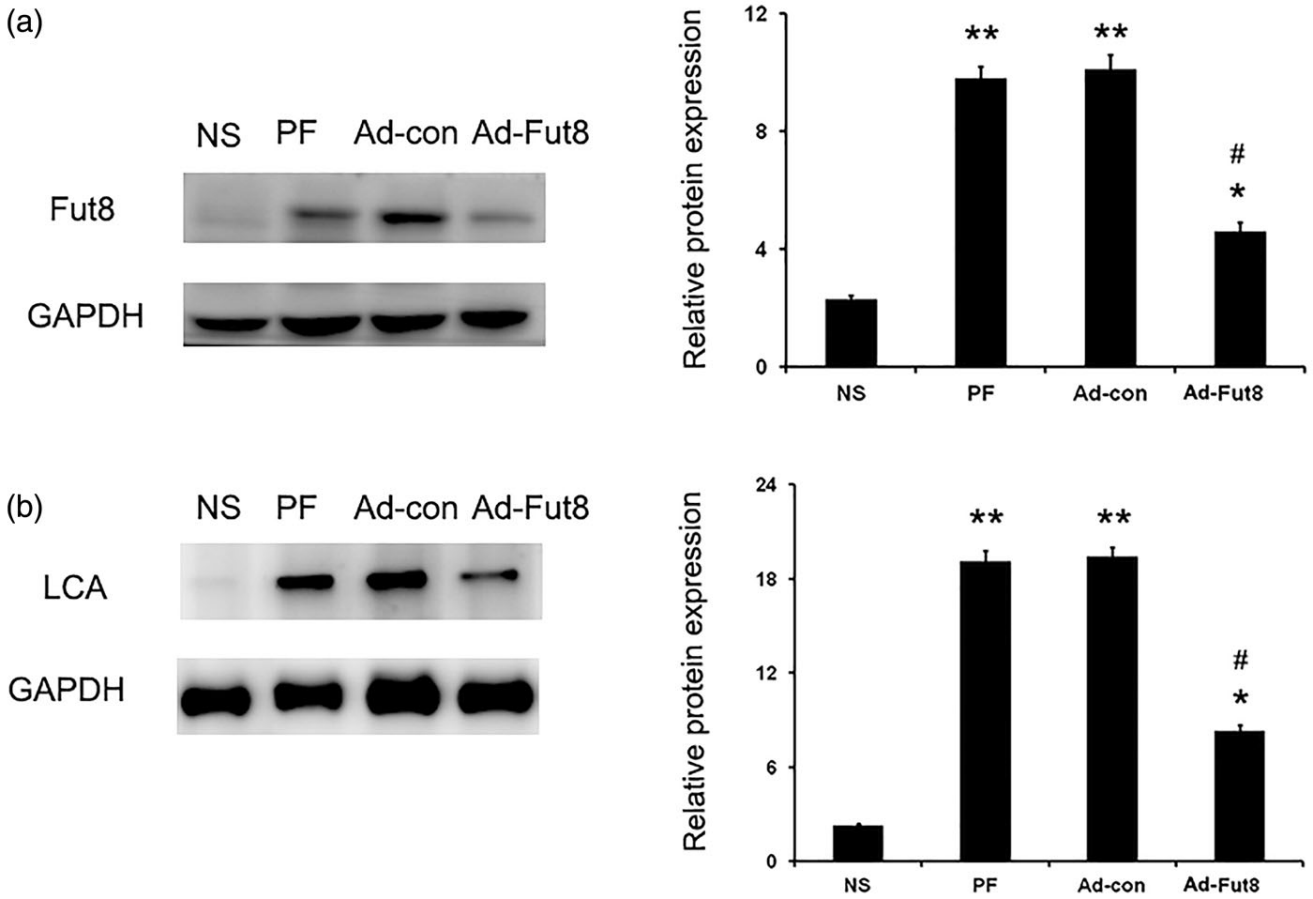
Fut8shRNA effectively inhibited Fut8 expression, and EGF receptor is modified by core fucosylation: (a) representative Western blot images and analyses of Fut8 in the normal saline (NS), peritoneal fibrosis (PF), adenovirus-control (Ad-con), and adenovirus-Fut8 (Ad-Fut8) groups; (b) representative Western and lectin blot analysis and quantification of the core fucose levels of the EGF receptor in the four groups. EGF receptor was immunoprecipitated from tissue lysates and then subjected to electrophoresis. After electroblotting, blots were probed by Lens culinaris agglutinin (LCA)–Biotin. Results are expressed as mean ± SEM of eight rats per group. *p < 0.05, **p < 0.01 vs. NS group. #*p* < 0.05 each group (except for NS group) vs. both PF and Ad-con group.

**Figure 3.**
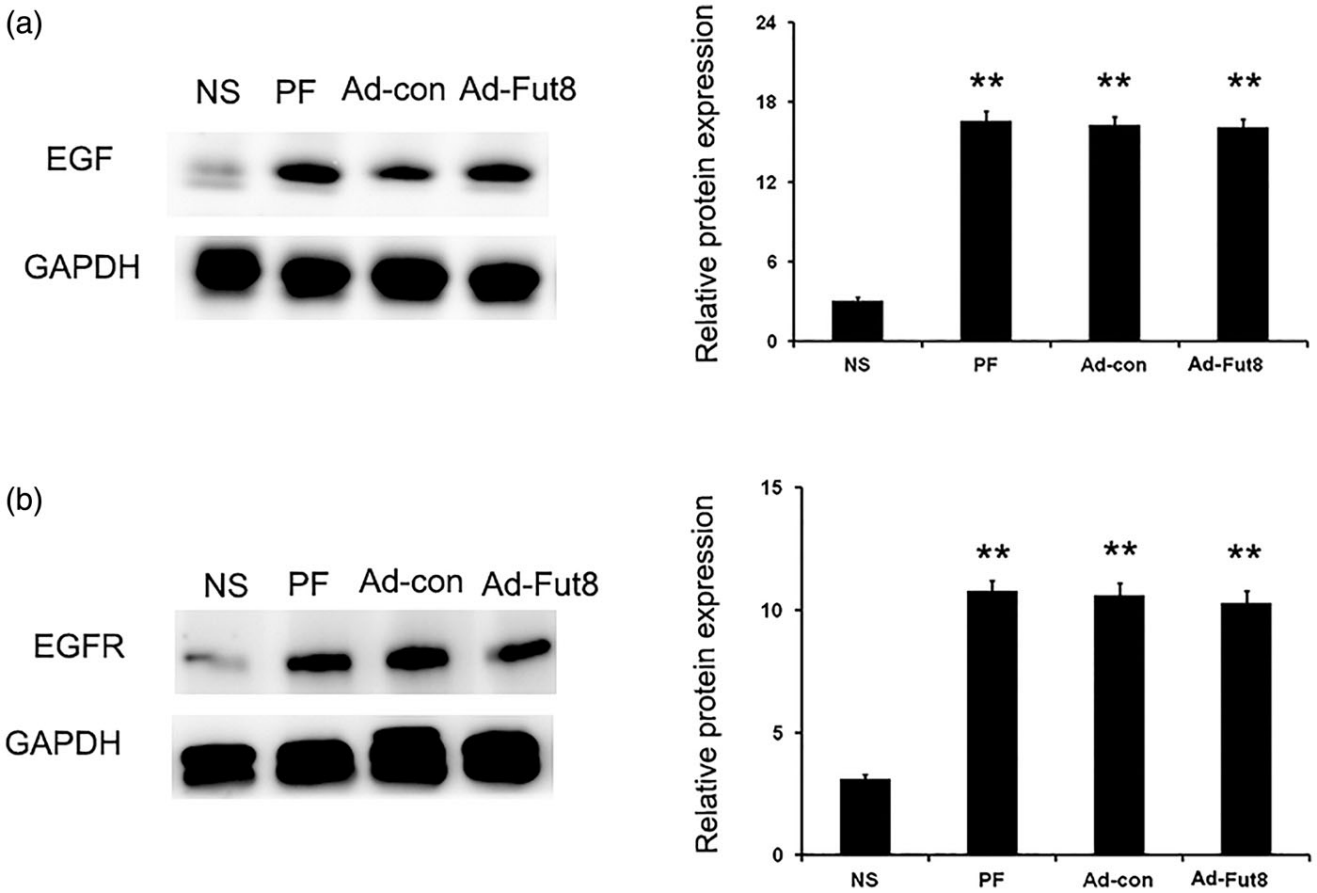
EGF and EGF receptor (EGFR) were upregulated by glucose PD dialysate. Representative Western blot images and analyses of EGF (a) and EGFR (b) in the normal saline (NS), peritoneal fibrosis (PF), adenovirus-control (Ad-con), and adenovirus-Fut8 (Ad-Fut8) groups. The expression of EGF and EGFR were not affected by Fut8shRNA presence. **p < 0.01 vs. NS group.

Next, we investigated whether Fut8 levels could be reduced by Fut8shRNA in the peritoneal membrane of rats. As shown in Figure 2(a), Fut8 expression was increased in the peritoneum of both PF and Ad-con rats; however, Fut8shRNA effectively inhibited Fut8 expression in the Ad-Fut8 group, indicating the successful generation of a Fut8-knockdown rat model of peritoneal fibrosis. We then examined whether EGF receptor was modified by core fucosylation. Positive bands that corresponded to core fucose were present after immunoprecipitation with EGF receptor (Figure 2(b)), demonstrating that the EGF receptor is modified by core fucoslyation. However, glucose PD dialysate upregulated the expression of EGF and EGF receptor, and Fut8shRNA had no effect on their expression levels (Figure 3).

### Fut8shRNA ameliorated glucose dialysate-induced peritoneal fibrosis

**Figure 4.**
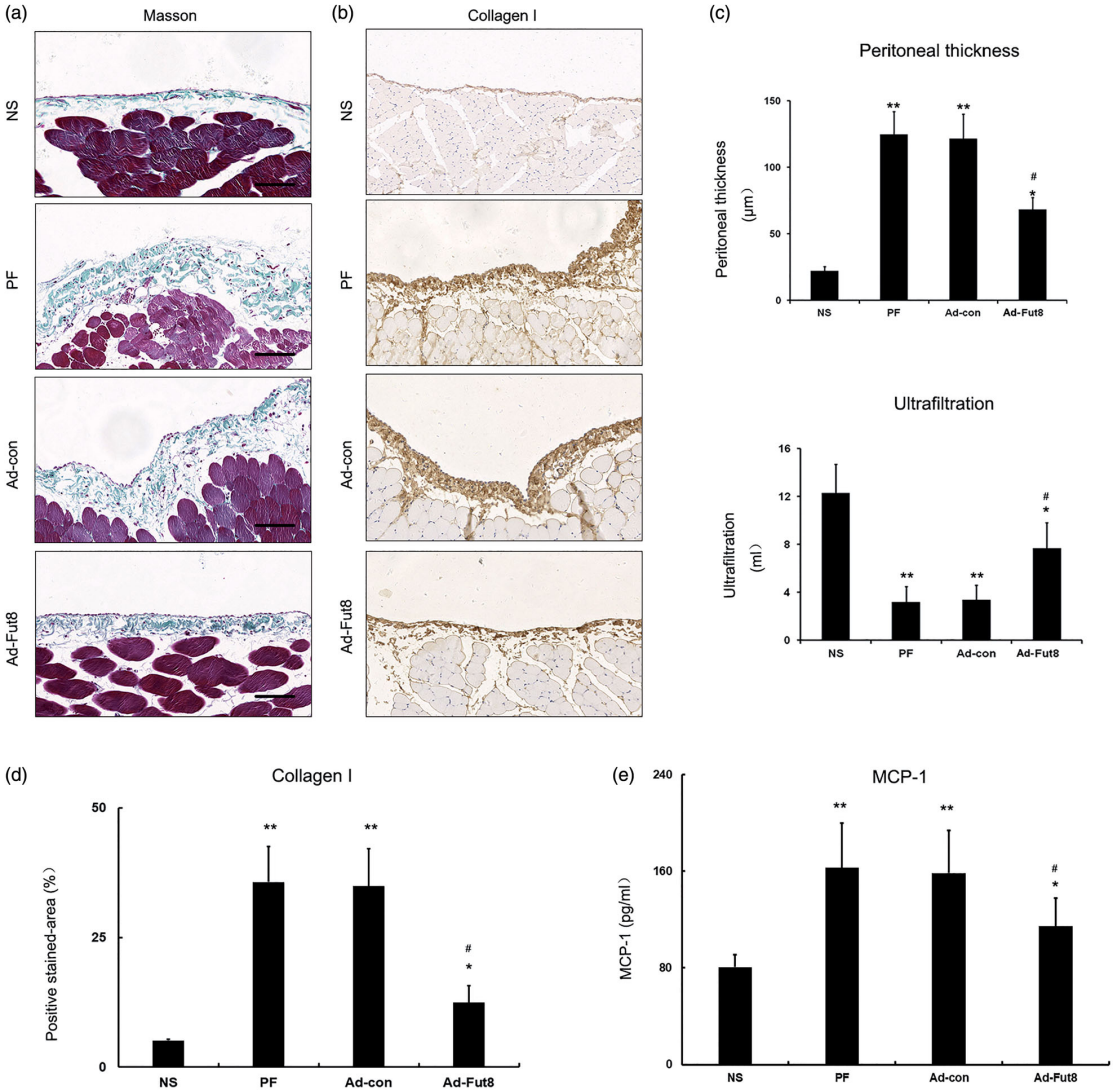
Fut8shRNA alleviated peritoneal structural and functional alteration and collagen I accumulation in rats with peritoneal fibrosis: (a) representative photographs of Masson’s staining (bar = 100 μm); (b) representative photographs of collagen I accumulation in peritoneal membrane (immunohischemistry, bar = 100 μm); (c) analysis of peritoneal thickness and ultrafiltration volume; (d) analysis of positive stained-area (%) of collagen I accumulation in peritoneal membrane; for quantitative assessment, an average of 5 independent measurements of positive stained - area (%) was calculated for each section using Image-Pro Plus 6.0 software; (e) analysis of MCP-1 in peritoneal effluent. Results are expressed as mean ± SEM of eight rats per group. *p < 0.05, **p < 0.01 vs. NS group. #*p* < 0.05 each group (except for NS group) vs. both PF and Ad-con group.

To investigate the effect of Fut8shRNA on the peritoneal membrane of rats, we examined the pathological changes by Masson’s staining and the functional changes by the peritoneal equilibrium test. Masson’s staining results showed an increase in peritoneal thickness in the PF and Ad-con groups (124.7 ± 17.3 μm, 121.6 ± 18.4 μm); however, peritoneal thickness was significantly decreased in Ad-Fut8 group (68.3 ± 9.1 μm, Figure 4(a,c)). The net ultrafiltration volume significantly decreased in PF and Ad-con groups (3.2 ± 1.3 mL, 3.4 ± 1.2 mL) compared to the NS group (12.3 ± 2.4 mL); whereas, net ultrafiltration volume was dramatically increased in the Ad-Fut8 group (7.7 ± 2.1 mL) compared to the PF and Ad-con groups (Figure 4(c)). We then examined the expression of collagen I in the peritoneal membrane by immunohistochemistry, and the results showed a significant decrease in the Ad-Fut8 group compared to the PF and Ad-con groups (Figure 4(b,d)). Next, the level of MCP-1 in the peritoneal effluent was also examined, and Fut8shRNA treatment significantly reduced MCP-1 level compared to PF and Ad-con groups (Figure 4(e), supplementary table 1).

### Fut8shRNA suppressed the phosphorylation of STAT3 and NF-κB in the peritoneal membrane of rats with peritoneal fibrosis

**Figure 5.**
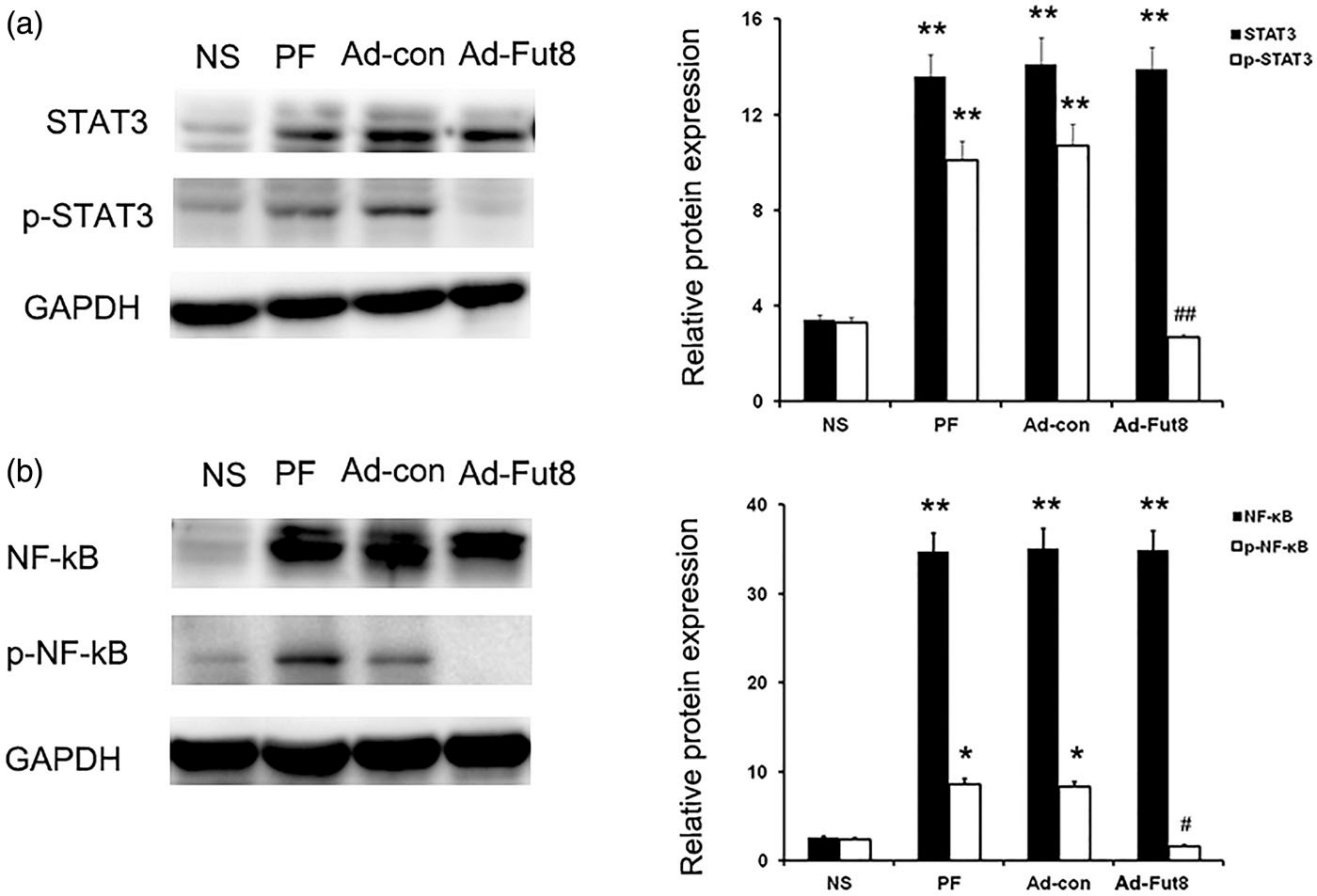
Fut8shRNA abolished activation of EGF signaling pathway. Representative western blot images and analyses of STAT3/p and STAT3, and NF -κB/p and NF-κB. *p < 0.05, **p < 0.01 vs. NS group. #*p* < 0.05, ##*p* < 0.01 each group (except for NS group) vs. both PF and Ad-con group.

**Figure 6.**
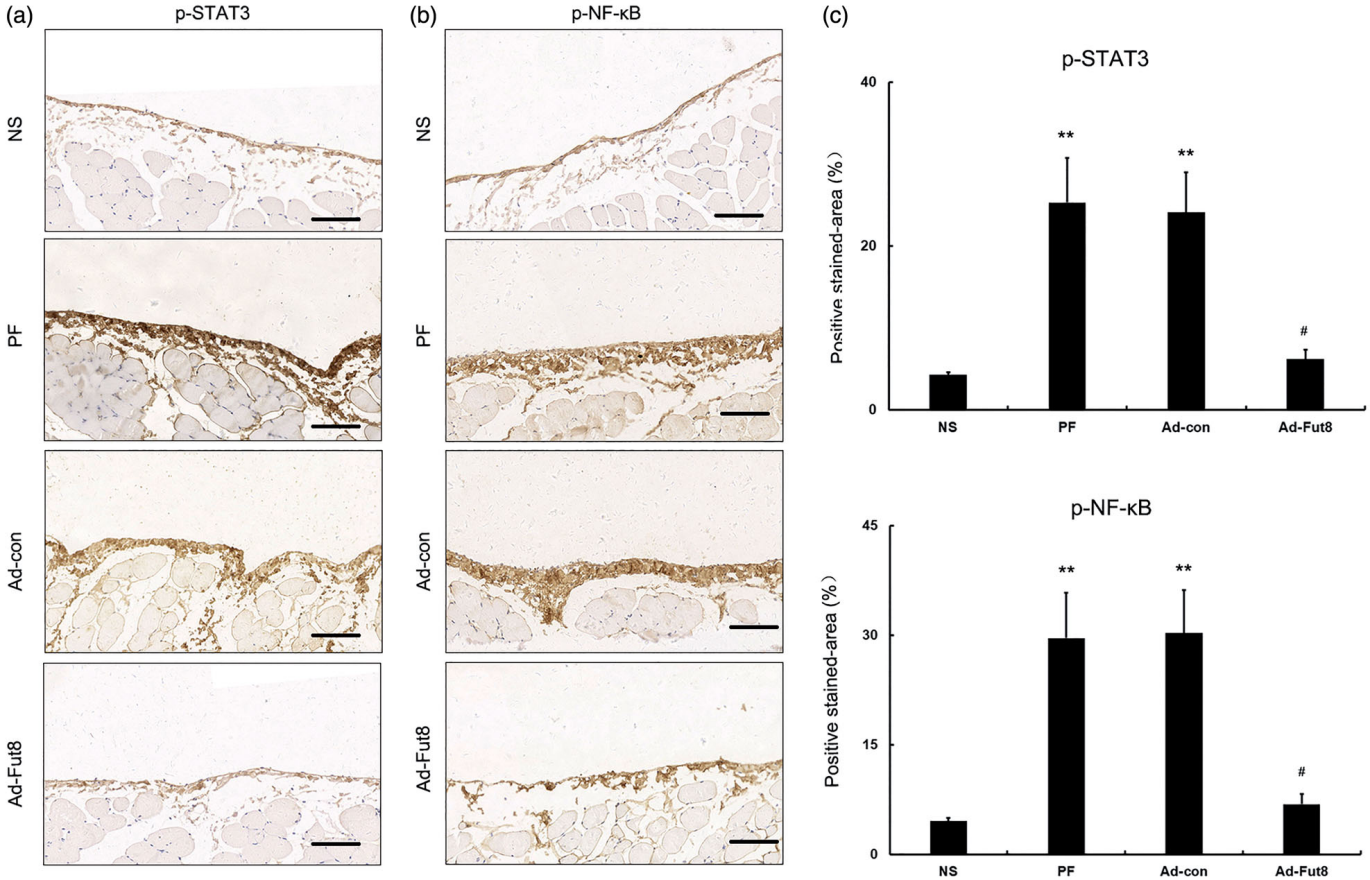
Fut8shRNA treatment reduced the expression of phosphorylation of STAT3 (p-STAT3) and NF-κB (p-NF-κB). **p < 0.01 vs. NS group. #*p* < 0.05 each group (except for NS group) vs. both PF and Ad-con group. Bar = 100 μm.

Since the EGF signaling pathway was shown to be involved in the progression of peritoneal fibrosis, we then studied EGF signaling activity after Fut8shRNA treatment. Western blot analysis showed that glucose peritoneal dialysate increased the expression of EGF and EGF receptor compared to the NS group, but Fut8shRNA treatment did not affect their expression levels (Figure 3). However, the expression of phosphorylation of STAT3 (p-STAT3) and NF-κB (p-NF-κB) was significantly decreased by Fut8shRNA treatment, despite their increased expression in PF and Ad-con rats (Figure 5). Next, the expression of phosphorylation of STAT3 (p-STAT3) and NF-κB (p-NF-κB) was also examined by immunohistochemistry, and Fut8shRNA treatment significantly reduced their expression compared to the PF and Ad-con groups (Figure 6). The above results demonstrate that Fut8shRNA could abolish EGF pathway activation without influencing the expression levels of EGF receptors, resulting in ameliorating peritoneal fibrosis.

## Discussion

The present study provides novel insights into the pathogenesis of peritoneal fibrosis by inhibiting core fucosylation of EGF receptor in rats with peritoneal fibrosis. Our findings showed that EGF receptor was modified by core fucosylation, and the level of core fucosylation of EGF receptor was upregulated in rats with peritoneal fibrosis. Inhibition of EGF receptor core fucosylation by Fut8shRNA treatment significantly ameliorated glucose dialysate-induced peritoneal fibrosis (pathological lesions and functional changes). A potential mechanism may be the inactivation of the EGF signaling pathway by decreasing the expression of p-STAT3 and p-NF-κB, but further investigation is required. This study further elucidates the mechanisms involved in peritoneal fibrosis progression, suggesting that core fucosylation of EGF receptors could be exploited as a novel therapeutic target for the peritoneal fibrosis treatment.

Currently, emerging evidence indicates that posttranslational glycosylation modifications of proteins play a key role in altering protein function, causing crucial effects on many important processes, such as cell growth, migration, and differentiation [20–23]. Our previous studies confirmed that blocking core fucosylation of TGF-β and PDGF receptors using Fut8shRNA could inactivate TGF-β and PDGF signaling pathways, attenuating peritoneal fibrosis in a rat model [17]. Since multiple signaling pathways are involved in the progression of peritoneal fibrosis and many key signaling proteins are modified by core fucosylation, we focused on the EGF signaling pathway in this study. In our experiments, the expression of core fucosylation of EGF receptor was markedly increased in rats with peritoneal fibrosis, suggesting core fucosylation of EGF receptor may play a role in peritoneal fibrosis To further elucidate the role of EGF receptor core fucosylation, we synthesized Fut8shRNA and confirmed that Fut8 was significantly knocked down in rats. And the core fucosylation of EGF receptor was also inhibited by Fut8shRNA. The results showed that Fut8shRNA treatment ameliorated glucose dialysate-induced peritoneal pathological lesions and functional changes. Therefore, Fut8shRNA may be a potential method of protecting the peritoneal membrane from glucose dialysate-induced fibrotic lesions.

We also observed the mechanism that inhibited core fucosylation of EGF receptor, which yielded protective effects on the peritoneal membrane. In this study, EGF receptor was first confirmed to be modified by core fucosylation, with protein expression levels upregulated in the peritoneal membrane of rats with peritoneal fibrosis. Interestingly, Fut8shRNA treatment suppressed the core fucosylation of EGF receptor but had no effect on its protein expression levels, indicating that the effects of core fucosylation of EGF receptors in peritoneal fibrosis were independent of their protein expression levels. Phosphorylation of STAT3 and NF-κB are markers of EGF signaling activation [24,25]. To determine whether core fucosylation of EGF receptor can regulate the activity of the EGF signaling pathway, phosphorylation of STAT3 and NF-κB were studied. Inhibition of the core fucosylation of EGF receptor was found to block their phosphorylation, showing decreasing EGF signaling activity by Fut8shRNA treatment. The protective effect of Fut8shRNA involves the inhibition of multiple signaling pathways, with the EGF signaling pathway being one example. However, our findings suggest that inhibition of core fucosylation of EGF receptor inactivates EGF signaling and ameliorates peritoneal fibrosis.

As inhibiting core fucosylation could suppress multiple signaling pathway activities (EGF, TGFβ, and PDGF signaling pathways), Fut8shRNA treatment may also inhibit TGFβ and PDGF signaling pathways and provide anti-fibrotic effects, and its protective effects may be stronger than a single signaling pathway inhibitor. This study is limited in that we only focused on EGF signaling pathway, and had no comparison between core fucosylation and EGF signaling inhibitors, which is crucial for understanding of the effect of multiple pathways inhibition of core fucosylation. Another limitation of our study is that vasculopathy and angiogenesis are also important lesions in the process of peritoneal fibrosis, and we did not perform such experiments. Since angiopoietin-Tie signaling is crucial for vasculopathy, and vascular endothelial-derived growth factor (VEGF) receptors are also fucosylated, so future research should be focused on vasculopathy and angiogenesis. In addition, EGF signaling pathway also plays an important role in angiogenesis [13], but we only focused on its effect on fibrotic changes in rats. Further studies are required to observe its effect on angiogenesis. Finally, encapsulating peritoneal sclerosis (EPS) is a distinct pathologic process from that of simple peritoneal fibrosis, and we did not perform experiments on EPS. Since preventive measures in intermediate stages between simple peritoneal fibrosis and EPS are crucial, further studies should focus on the intermediate stages by inhibition of core fucosylation.

## Conclusions

Our study shows that there are increased levels of EGF receptor core fucosylation in rats with peritoneal fibrosis, and its inhibition by Fut8shRNA ameliorated fibrotic changes. The mechanism may be that inhibition of core fucosylation of the EGF receptor inactivates the EGF signaling pathway by suppressing the phosphorylation of STAT3 and NF-κB in the peritoneal membrane of rats with peritoneal fibrosis, although further investigation is required.

## Supplementary Material

Supplemental Material

Supplemental Material
