# Supplementary figures and images for: Blocking core fucosylation of epidermal growth factor (EGF) receptor prevents peritoneal fibrosis progression
Source: Ren Fail. 2021 May 17;43(1):869–77. doi: 10.1080/0886022X.2021.1918557 (PMC8143636; doi:10.1080/0886022X.2021.1918557)

(a)

Collagen I

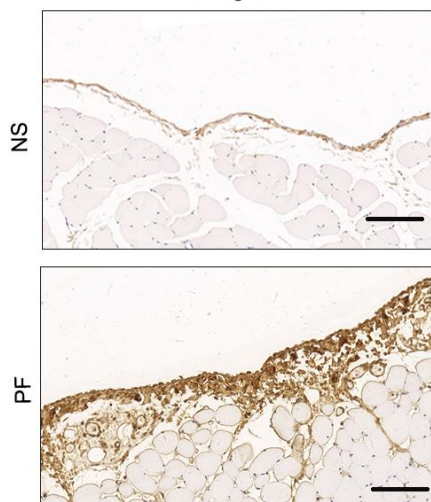

(b)

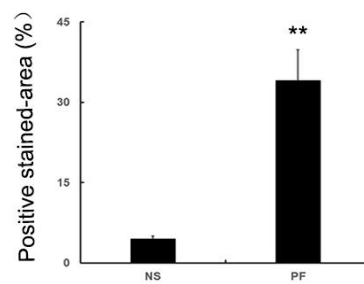

Supplement: Supplemental Material [file IRNF_A_1918557_SM7988.pdf]
