# Supplementary material for: Blocking core fucosylation of epidermal growth factor (EGF) receptor prevents peritoneal fibrosis progression
Source: Ren Fail. 2021 May 17;43(1):869–77. doi: 10.1080/0886022X.2021.1918557 (PMC8143636; doi:10.1080/0886022X.2021.1918557)
Supplement: Supplemental Material [file IRNF_A_1918557_SM7980.pdf]

Supplementary table

Table 1. The levels of MCP-1 in peritoneal effluent.

NS	PF	Ad-con	Ad-Fut8
80.7±10.2	162.9±37.2	158.4±35.4	114.4±23.5

MCP-1: monocyte chemoattractant protein-1; NS: normal saline; PF: peritoneal fibrosis; Ad-con: adenovirus-control; Ad-Fut8: adenovirus-Fut8.
